# Supplementary material for: Safety Monitoring of COVID-19 Vaccines in Persons with Prior SARS-CoV-2 Infection: A European Multi-Country Study
Source: Vaccines (Basel). 2024 Feb 26;12(3):241. doi: 10.3390/vaccines12030241 (PMC10974422; doi:10.3390/vaccines12030241)
Supplement: Supplementary file 1 [file vaccines-12-00241-s001.zip › vaccines-2835659-supplementary.pdf]

Supplementary Figure S1: Heatmaps with the frequency of reported local solicited ADRs following the first, second and the booster dose of any vaccine in people with SARS-CoV-2 infection vs. matched control, stratified by gender

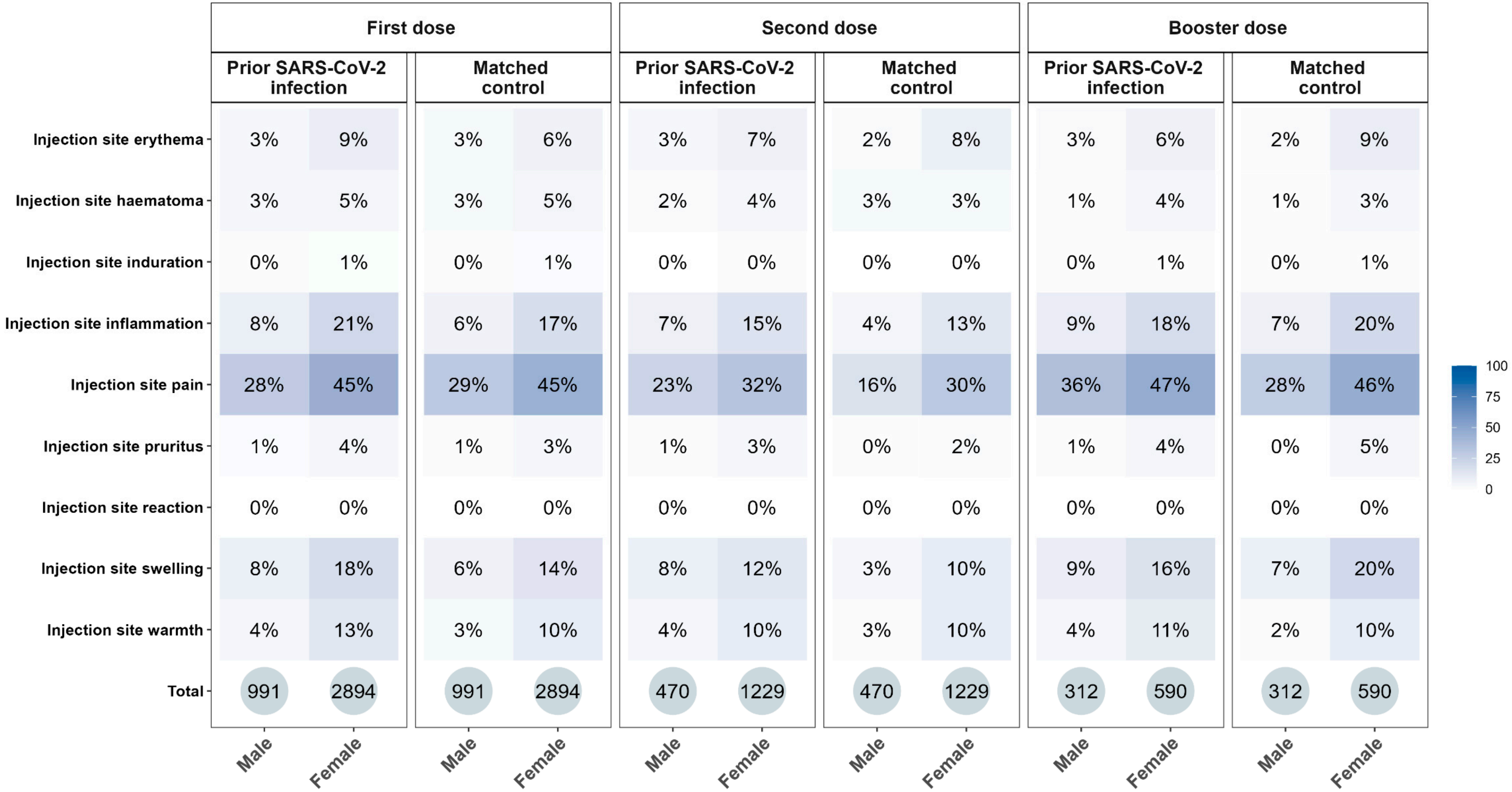

Supplementary Figure S2: Heatmaps with the frequency of reported systemic solicited ADRs following the first, second and the booster dose of any vaccine in people with SARS-CoV-2 infection vs. matched control, stratified by gender

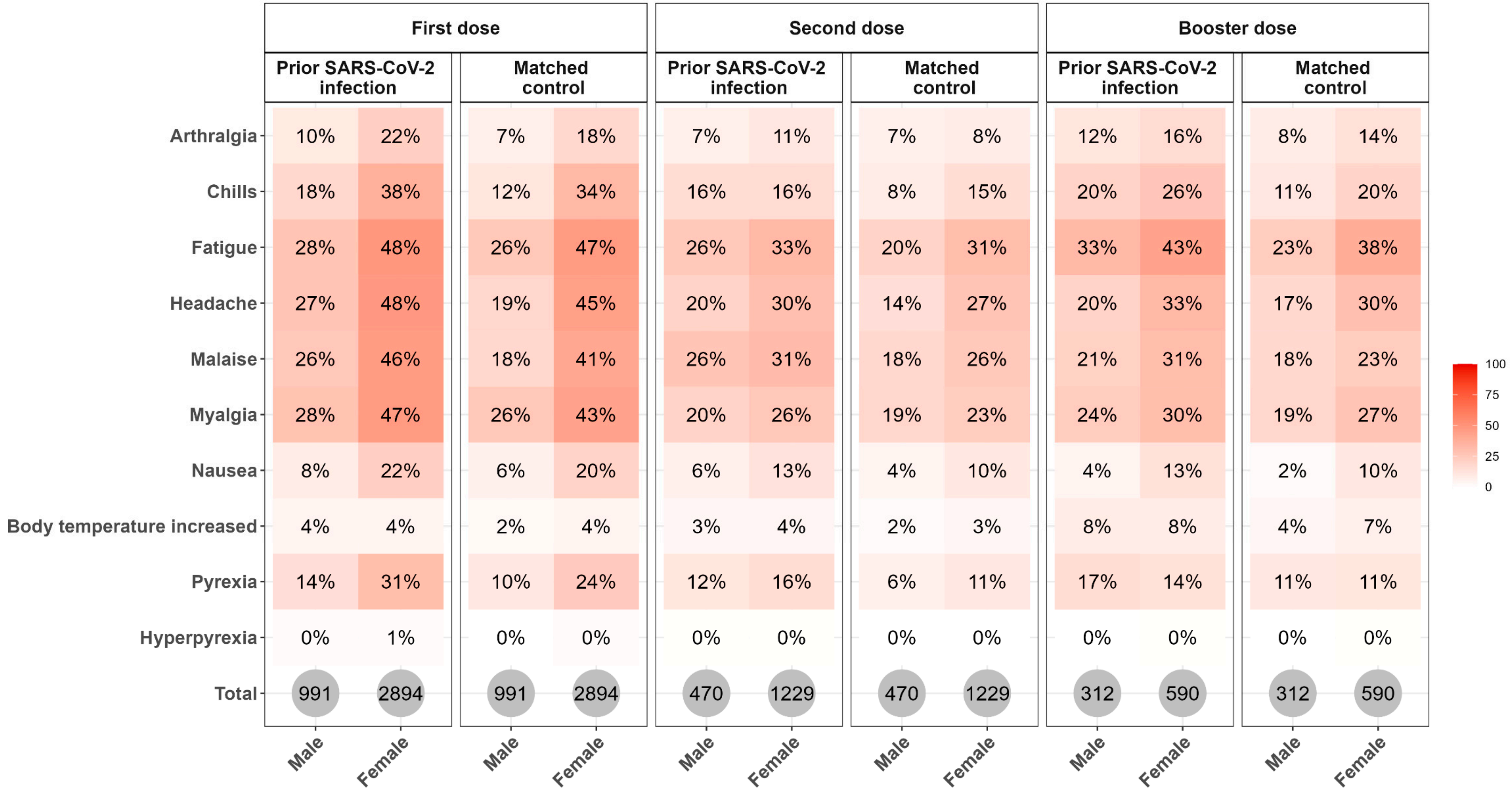

Supplementary Figure S3: Forest plot relating medical history to the occurrence of at least one ADR in people with prior SARS-CoV-2 infection for the first and the booster dose

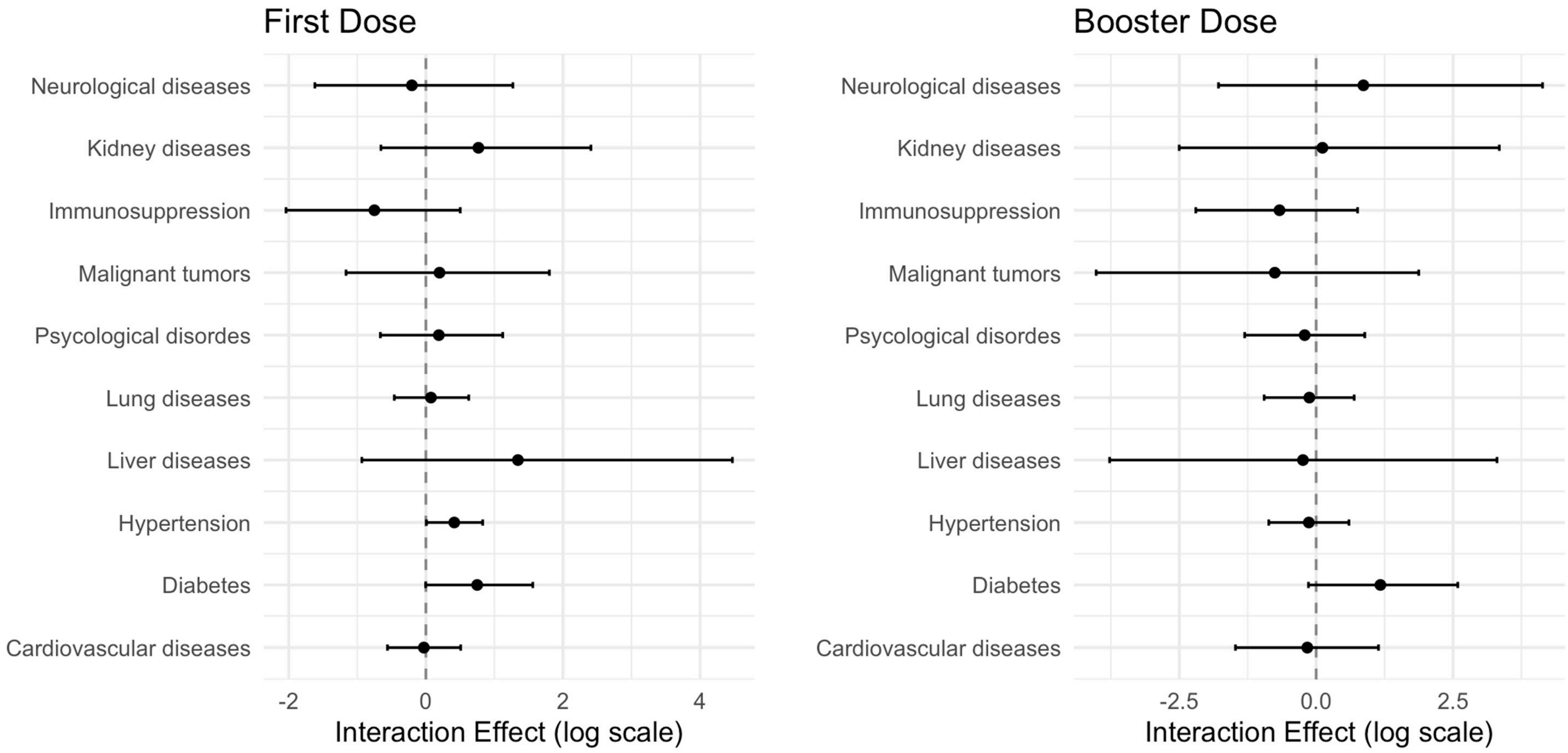

Supplementary Table S1: Experimental and observational studies related to COVID-19 vaccines in clinicaltrials.gov and PubMed, as of November 21, 2023

Study title	NCT	Phase	Study Design	Study Status	Intervention/treatment	Study population	Primary outcome	Main findings
**Clinical trials**								
BNT162b2 Vaccination With 2 Doses in COVID-19 Negative Volunteers and With a Single Dose in COVID-19 Positive Volunteers (CoviCompareP)	NCT04824638	II	Interventional, Non-Randomized	Active, not recruiting	Comirnaty (BioNTech/Pfizer) - BNT162b2	267 participants	To evaluate IgG humoral response to vaccine 28 days post vaccination	NA
Factors Influencing the COVID-19 Vaccine Immune Response According to Age and Presence or Not of a Past History of COVID-19 (COVIMMUNAGE)	NCT05047718	IV	Interventional, Non-Randomized	Completed	Comirnaty (BioNTech/Pfizer) - BNT162b2<br>Spikevax (mRNA-1273) - mRNA-1273	54 participants	To evaluate Anti-S neutralizing antibody titer	NA
**Observational studies**								
Adverse Events Following Immunization With mRNA and Viral Vector Vaccines in Individuals With Previous Severe Acute Respiratory Syndrome Coronavirus 2 Infection From the Canadian National Vaccine Safety Network	N.A.	N.A.	Prospective, multicenter, cohort study	Completed	Comirnaty (BioNTech/Pfizer) - BNT162b2<br>Spikevax (mRNA-1273) - mRNA-1273<br>Vaxzevria (ChAdOx1-S) - ChAdOx1-S	684,998 participants	To profile common AEFI within 7 days after the first, second, and third doses of COVID-19 vaccine in participants with and without SARS-CoV-2 infection	After dose 1, individuals with moderate (bedridden) to severe (hospitalized) COVID-19 who received BNT162b2, mRNA-1273, or ChAdox1-S vaccines had higher odds of a health event preventing daily activities, resulting in work absenteeism or requiring medical consultation. Following dose 2 and 3, the greater risk associated with previous infection was also present but was attenuated compared with dose 1.
An Analysis of SARS-CoV-2 Vaccine Reactogenicity: Variation by Type, Dose, and History, Severity, and Recency of Prior SARS-CoV-2 Infection	N.A.	N.A.	Longitudinal cohort study	Completed	Janssen - Ad26.COV2.S<br>Spikevax (mRNA-1273) - mRNA-1273<br>Comirnaty (BioNTech/Pfizer) - BNT162b2	2,789 participants	To characterize partvaccine reactogenicity as a function of prevaccination SARS-CoV-2 infection vaccine type and dose, and demographic factors	Severe side effects were most common with the Ad26.COV2.S (Janssen/Johnson and Johnson) vaccine, followed by mRNA-1273 (Spikevax (mRNA-1273)) then BNT162b2 (Comirnaty (BioNTech/Pfizer)/BioNTech). Severe side effects were more common after the second than first dose (11% vs 4%; *P* < .001).

								First (but not second) dose side effects were more common in those with vs without prior severe acute respiratory syndrome coronavirus 2 infection.
Association between previous infection with SARS CoV-2 and the risk of self-reported symptoms after mRNA BNT162b2 vaccination: Data from 3,078 health care workers	N.A.	N.A.	Survey study	Completed	Comirnaty (BioNTech/Pfizer) - BNT162b2	3,078 participants	To verify whether a previous SARS CoV-2 infection or disease brings about an increased risk of suffering local or systemic symptoms upon anti-SARS CoV-2 vaccination	Previous SARS-CoV-2 infection/COVID-19 occurred in 396 subjects (12·9%). 59·6% suffered from ≥1 local or systemic symptom after the first and 73·4% after the second dose. MSS occurred in 6·3% of cases (14·4% with previous vs 5·1% with no COVID-19 $p<0·001$) and in 28·3% (24·5% in COVID-19 vs 28·3% no COVID, $p = 0·074$) after the first and second dose, respectively.
BNT162b2 mRNA COVID-19 vaccine Reactogenicity: The key role of immunity	NCT04402827	N.A.	Prospective, Case-Control study	Completed	Diagnostic Test	140 participants	To establish differences in susceptibility to SARS CoV-2 infection among health care workers (HCW) highly exposed to patients with COVID-19 diagnosis	BNT162b2 vaccine reactogenicity after first dose is attributable to pre-existing cellular immunity elicited by prior COVID-19 or cross-reactivity. Reactogenicity following second dose suggests an immunity-boosting effect. (Vizcarra, 2021)
Evaluation of Side Effects Associated with COVID-19 Vaccines in Saudi Arabia	N.A.	N.A.	Retrospective, cross-sectional study	Completed	Comirnaty (BioNTech/Pfizer) - BNT162b2 Vaxzevria (ChAdOx1-S) - ChAdOx1-S	515 participants	To evaluated the short-term side effects after receiving either Comirnaty (BioNTech/Pfizer)- BioNTech mRNA (BNT162b2) or Oxford-Vaxzevria (ChAdOx1-S) (ChAdOx1 nCoV-19) vaccines	Side effects associated with COVID-19 vaccines have been reported by 60% of the study subjects, and most of them reported fatigue (90%), pain at the site of the injections (85%)

Impact of prior SARS-CoV-2 infection on incidence of hospitalization and adverse events following mRNA SARS-CoV-2 vaccination: A nationwide, retrospective cohort study	N.A.	N.A.	Retrospective cohort study	Completed	Comirnaty (BioNTech/Pfizer) - BNT162b2<br>Spikevax (mRNA-1273) - mRNA-1273	102,829 participants with previous SARS-CoV-2 infection	To evaluate the impact of prior SARS-CoV-2 infection on outcomes shortly after vaccination using a longitudinal design	DOI: 10.1016/j.vaccine.2022.01.026
Increased adverse events following third dose of BNT162b2/Comirnaty (BioNTech/Pfizer) vaccine in those with previous COVID-19, but not with concurrent influenza vaccine	N.A.	N.A.	Retrospective, descriptive study	Completed	Comirnaty (BioNTech/Pfizer) - BNT162b2	534 participants	To explore the impact of risk factors for vaccine-associated AEs following the third/booster dose of BNT162b2/Comirnaty (BioNTech/Pfizer) vaccine in healthcare workers	For all doses of BNT162b2/Comirnaty (BioNTech/Pfizer) vaccine there was a cluster of systemic AEs that were consistently worse in HCWs with a prior history of COVID-19. AEs were no worse in HCWs who received their third/booster dose within 7 days of the influenza jab, rather than further apart
Reactogenicity of COVID-19 Vaccines in Patients With a History of COVID-19 Infection: A Survey Conducted in Pakistan	N.A.	N.A.	Comparative cross-sectional study	Completed	Comirnaty (BioNTech/Pfizer) - BNT162b2<br>Spikevax (mRNA-1273) - mRNA-1273<br>Vaxzevria (ChAdOx1-S) - ChAdOx1-S<br>CanSino - Convidecia	421 participants	To analyze the reactogenicity and safety profiles of different types of vaccines available in Pakistan	0.7% of the individuals reported experiencing serious adverse effects. Injection site pain (35.9%) was noted to be the most remarkable post-vaccination side effect followed by fever (33.2%) and fatigue (23.1%). Prior COVID-19 infection was not associated with the severity of any COVID-19 vaccine-related side effect ($p > 0.05$), except dyspnea. Younger participants and the female gender were substantially linked to post-vaccination adverse effects.
Previous COVID-19 infection but not Long-COVID is associated with increased adverse events following BNT162b2/Pfizer vaccination	N.A.	N.A.	survey-based observational study	Completed	Comirnaty (BioNTech/Pfizer) - BNT162b2	974 participants	To establish whether individuals with prior history of COVID-19 were more likely to experience AEs after BNT162b2/Pfizer vaccination, than those without previous COVID-	Prior COVID-19 infection, but not Long-COVID, were associated with increased risk of self-reported AEs adverse events following BNT162b2/Pfizer vaccination

							19, and whether COVID-19 vaccination interval influenced AE severity	
Prior COVID-19 infection is associated with increased Adverse Events (AEs) after the first, but not the second, dose of the BNT162b2/Comirnaty (BioNTech/Pfizer) vaccine	N.A.	N.A.	survey-based observational study	Completed	Comirnaty (BioNTech/Pfizer) - BNT162b2	2,146 participants	To explore severity and duration of AEs after first and second doses of BNT162b2/Comirnaty (BioNTech/Pfizer) vaccination	Post-dose AEs were worse in those with prior COVID-19 after the first, but not the second dose of vaccine. Second dose AEs were greater in frequency/severity, regardless of COVID-19 history, and they were more systemic in nature. Women and younger HCW were more likely to report AEs after both doses, while dosing interval had no effect on AEs.
Post-Covid-19-vaccination adverse events and healthcare utilization among individuals with or without previous SARS-CoV-2 infection	N.A.	N.A.	Retrospective, cohort study	Completed	Comirnaty (BioNTech/Pfizer) - BNT162b2<br>Sinovac - CoronaVac	3,922 participants with previous SARS-CoV-2 infection	To detect any differences in the risk of adverse events of special interest (AESI), accident and emergency room (A&E) visit, and hospitalization between those with a previous infection and those without following vaccination	No significant association was observed between previous SARS-CoV-2 infection and AESI or hospitalization. Previous SARS-CoV-2 infection was significantly associated with a lower risk of A&E visit (CoronaVac: hazard ratios [HR] = 0.56, 95% confidence intervals [CI]: 0.32–0.99; Comirnaty: HR = 0.62, 95% CI: 0.47–0.82).
Safety of Pfizer-BioNTech vaccine in a cohort of healthcare providers: Differences between naïve and previously infected by SARS-CoV-2	N.A.	N.A.	Retrospective cohort study	Completed	Comirnaty (BioNTech/Pfizer)	406 participants	To compared the incidence of AEs and compared individuals with 0–3 different AEs to those with 4 or more AEs. The relative risks (RR) and their 95% confidence intervals were calculated.	Past infection was associated with having more AEs after the first dose ($p < 0.001$), but not the second one ($p = 0.476$), as well as a higher incidence of AEs ($p < 0.001$). Common AEs that were statistically associated with past COVID infection included arthralgia, asthenia, fever, chills, headache, and myalgia ($p \leq 0.001$).
Self-Reported Real-World Safety and Reactogenicity of	N.A.	N.A.	Online survey	Completed	Comirnaty (BioNTech/Pfizer) - BNT162b2	2002 participants	To compare the safety profiles of available COVID-19 vaccines and	A prior COVID-19 infection was associated with an 8% increase in the risk of having any side

COVID-19 Vaccines: A Vaccine Recipient Survey					Vaxzevria (ChAdOx1-S) - ChAdOx1-S		evaluate their side effects in different groups of vaccine recipients.	effects after the first vaccine dose
Vaccine side-effects and SARS-CoV-2 infection after vaccination in users of the COVID Symptom Study app in the UK: a prospective observational study	N.A.	N.A.	Prospective	Completed	Comirnaty (BioNTech/Pfizer) - BNT162b2 Vaxzevria (ChAdOx1-S) - ChAdOx1-S	627,383 participants	To investigate the adverse effects and infection rate of vaccinated people in a community (general population app users) scenario	Systemic side-effects were reported by 13·5% (38 155 of 282 103) of individuals after the first dose of BNT162b2, by 22·0% (6216 of 28 207) after the second dose of BNT162b2, and by 33·7% (116 473 of 345 280) after the first dose of ChAdOx1 nCoV-19. Systemic side-effects were more common among individuals with previous SARS-CoV-2 infection than among those without known past infection.
Vaccine Side Effects Following COVID-19 Vaccination Among the Residents of the UAE—An Observational Study	N.A.	N.A.	Cross-sectional study	Completed	Sinopharm - BBIBP-CorV Comirnaty (BioNTech/Pfizer) - BNT162b2 Vaxzevria (ChAdOx1-S) - ChAdOx1-S	1,878 participants	To identify the side effects reported after receiving a COVID-19 vaccination	The major adverse effects reported by the COVID-19 vaccine recipients were pain at the site of injection, fatigue and drowsiness, and headache followed by joint/muscle pain. The adverse effects were more common among recipients of mRNA Comirnaty (BioNTech/Pfizer) vaccine than among recipients of inactive Sinopharm

Abbreviations: N.A. = not applicable

Supplementary Table S2. Frequency of reported local and systemic solicited ADRs following the first dose, stratified by vaccine brands, in people with SARS-CoV-2 infection vs. matched control

	First dose											
	Vaxzevria (ChAdOx1-S) N= 1,410 (%)			Comirnaty (BioNTech/Pfizer) N= 1,372 (%)			Spikevax (Moderna) N= 668 (%)			Jcovden (Ad26.COV2-S) N= 422 (%)		
	Prior SARS-CoV-2 infection	Matched control	p-value	Prior SARS-CoV-2 infection	Matched control	p-value	Prior SARS-CoV-2 infection	Matched control	p-value	Prior SARS-CoV-2 infection	Matched control	p-value
At least one ADR, n (%)	1371 (97.2)	1337 (94.8)	<0.001**	1080 (78.7)	967 (70.5)	<0.001*	637 (95.6)	612 (91.6)	0.067	382 (90.5)	356 (84.4)	0.812
At least one solicited ADR, n (%)	1187 (84.2)	1286 (91.2)	<0.001**	752 (54.8)	788 (57.4)	0.724	463 (45.1)	515 (77.1)	0.659	372 (88.2)	348 (82.5)	0.569
**Local solicited ADR (MedDRA PT)**												
Injection site erythema	128 (9.1)	107 (7.6)	0.173	51 (3.7)	29 (2.1)	0.010*	75 (8.4)	60 (9)	0.530	26 (6.2)	10 (2.4)	<0.001*
Injection site haematoma	73 (5.2)	82 (5.8)	0.509	37 (2.7)	39 (2.8)	0.869	27 (2.5)	32 (4.8)	0.002*	27 (6.4)	12 (2.8)	<0.001*
Injection site induration	8 (0.6)	16 (1.1)	0.151	7 (0.5)	6 (0.4)	<0.001*	9 (0.1)	19 (2.8)	<0.001*	3 (0.7)	1 (0.2)	<0.001*^
Injection site inflammation	324 (23)	279 (19.8)	0.043*	138 (10.1)	112 (8.2)	0.007*	145 (14.7)	127 (19)	0.219	77 (18.2)	33 (7.8)	0.007*
Injection site pain	695 (49.3)	676 (47.9)	0.498	433 (31.6)	440 (32.1)	0.88	285 (23.4)	324 (48.5)	0.43	179 (42.4)	136 (32.2)	0.140
Injection site pruritus	66 (4.7)	51 (3.6)	0.186	21 (1.5)	21 (1.5)	0.975	33 (2.7)	33 (4.9)	0.991	7 (1.7)	5 (1.2)	0.365
Injection site reaction	1 (0.1)	1 (0.1)	1	2 (0.1)	1 (0.1)	<0.001*^	-	1 (0.1)	0.616	-	-	-
Injection site swelling	284 (20.1)	231 (16.4)	0.011*	116 (8.5)	94 (6.9)	<0.001*	134 (11.8)	109 (16.3)	0.021	69 (16.4)	36 (8.5)	0.063
Injection site warmth	202 (14.3)	170 (12.1)	0.084	88 (6.4)	63 (4.6)	0.244	97 (10.2)	80 (12)	0.603	34 (8.1)	15 (3.6)	0.244
**Systemic solicited ADR (MedDRA PT)**												
Arthralgia	427 (30.3)	382 (27.1)	0.067	102 (7.4)	75 (5.5)	<0.001*	95 (10.8)	48 (7.2)	0.009*	114 (27)	82 (19.4)	<0.001*
Chills	765 (54.3)	793 (56.2)	0.307	161 (11.7)	67 (4.9)	<0.001*	161 (18.3)	53 (7.9)	<0.001*	186 (44.1)	173 (41)	0.809
Fatigue	796 (56.5)	860 (61)	0.016*	372 (27.1)	330 (24.1)	0.593	259 (27.5)	213 (31.9)	0.320	248 (58.8)	213 (50.5)	0.667
Headache	851 (60.4)	901 (63.9)	0.057	317 (23.1)	219 (16)	0.037*	211 (22.9)	140 (21)	0.006*	254 (60.2)	217 (51.4)	0.565
Malaise	834 (59.1)	878 (62.3)	0.097	291 (21.2)	162 (11.8)	<0.001*	240 (27.8)	130 (19.5)	<0.001*	232 (55)	191 (45.3)	0.452
Myalgia	768 (54.5)	793 (56.2)	0.363	378 (27.6)	335 (24.4)	0.602	270 (23.4)	201 (30.1)	0.115	226 (53.6)	171 (40.5)	0.101
Nausea	388 (27.5)	390 (27.7)	0.966	129 (9.4)	92 (6.7)	<0.001*	112 (12.6)	67 (10)	<0.001*	100 (23.7)	88 (20.9)	<0.001*
Body_temperature_increased	72 (5.1)	95 (6.7)	0.079	38 (2.8)	8 (0.6)	0.025*	28 (4.2)	16 (2.4)	<0.001*	20 (4.7)	33 (7.8)	0.007*
Pyrexia	625 (44.3)	600 (42.6)	0.362	109 (7.9)	50 (3.6)	<0.001*	137 (18.3)	26 (3.9)	<0.001*	165 (39.1)	125 (29.6)	0.011*
Hyperpyrexia	18 (1.3)	12 (0.9)	0.359	-	1 (0.1)	0.617	1 (0.1)	-	1^	3 (0.7)	2 (0.5)	0.038*^
At least one unsolicited ADR, n (%)	1128 (80.0)	1031 (73.1)	0.006*	447 (32.6)	383 (27.9)	0.373	393 (58.8)	271 (40.6)	0.021*	292 (69.2)	248 (58.8)	0.310
At least one AESI, n (%)	1 (0.1)	3 (0.2)	<0.001*^	2 (0.1)	3 (0.2)	0.045*^	1 (0.1)	1 (0.1)	1^			-
At least one serious ADR, n (%)	2 (0.1)	3 (0.2)	0.045*^	7 (0.5)	2 (0.1)	<0.001*^	4 (0.6)	4 (0.6)	0.856^		1 (0.2)	0.606

Abbreviations: ADR = adverse drug reaction, MedDRA=Medical Dictionary for Regulatory Activities; PT=preferred term; * = statistically significant; ^= Fisher's exact test
13 vaccinees who reported an unknown vaccine brand and 1 Novavax were excluded

Supplementary Table S3. Frequency of reported local and systemic solicited ADRs following the second dose, stratified by vaccine brands, in people with SARS-CoV-2 infection vs. matched control

	Second dose								
	Vaxzevria (ChAdOx1-S) N= 646 (%)			Comirnaty (BioNTech/Pfizer) N= 707 (%)			Spikevax (Moderna) N= 344 (%)		
	Prior SARS-CoV-2 infection	Matched control	p-value	Prior SARS-CoV-2 infection	Matched control	p-value	Prior SARS-CoV-2 infection	Matched control	p-value
At least one ADR, n (%)	613 (97.7)	634 (98.1)	<0.001*	491 (69.4)	567 (80.2)	0.585	339 (98.5)	323 (93.9)	0.004*
At least one solicited ADR, n (%)	334 (51.7)	306 (47.4)	0.639	397 (56.2)	323 (45.7)	0.246	301 (87.5)	297 (86.3)	0.868
**Local solicited ADR (MedDRA PT)**									
Injection site erythema	21 (3.3)	25 (3.9)	0.549	24 (3.4)	28 (4)	0.538	56 (16.3)	55 (16)	1
Injection site haematoma	20 (3.1)	14 (2.2)	0.016*	18 (2.5)	16 (2.3)	0.615	17 (4.9)	18 (5.2)	0.596
Injection site induration	1 (0.2)	-	1^	3 (0.4)	1 (0.1)	<0.001*^	1 (0.3)	1 (0.3)	1^
Injection site inflammation	64 (9.9)	43 (6.7)	0.197	57 (8.1)	48 (6.8)	0.400	98 (28.5)	86 (25)	0.849
Injection site pain	159 (24.6)	136 (21.1)	0.389	183 (25.9)	146 (20.7)	0.211	156 (45.3)	156 (45.3)	0.846
Injection site pruritus	9 (1.4)	5 (0.8)	<0.001*	16 (2.3)	5 (0.7)	<0.001*	18 (5.2)	20 (5.8)	<0.001*
Injection site reaction	-	-	-	-	-	-	-	-	-
Injection site swelling	54 (8.4)	38 (5.9)	0.293	57 (8.1)	35 (5)	0.045*	79 (23)	62 (18)	<0.001*
Injection site warmth	37 (5.7)	22 (3.4)	0.014*	32 (4.5)	37 (5.2)	0.713	68 (19.8)	79 (23)	0.574
**Systemic solicited ADR (MedDRA PT)**									
Arthralgia	35 (5.4)	27 (4.2)	0.196	61 (8.6)	37 (5.2)	0.061	72 (20.9)	65 (18.9)	0.903
Chills	55 (8.5)	44 (6.8)	0.540	98 (13.9)	48 (6.8)	0.004*	122 (35.5)	131 (38.1)	<0.001*
Fatigue	149 (23.1)	138 (21.4)	0.886	193 (27.3)	150 (21.2)	0.144	184 (53.5)	182 (52.9)	0.947
Headache	143 (22.1)	123 (19)	0.112	166 (23.5)	119 (16.8)	<0.001*	153 (44.5)	157 (45.6)	<0.001*
Malaise	120 (18.6)	94 (14.6)	<0.001*	200 (28.3)	118 (16.7)	<0.001*	186 (54.1)	184 (53.5)	<0.001*
Myalgia	102 (15.8)	84 (13)	<0.001*	153 (21.6)	130 (18.4)	0.366	156 (45.3)	160 (46.5)	<0.001*
Nausea	33 (5.1)	37 (5.7)	0.632	72 (10.2)	36 (5.1)	0.008*	84 (24.4)	74 (21.5)	0.804
Body_temperature_increased	11 (1.7)	9 (1.4)	<0.001*	24 (3.4)	12 (1.7)	<0.001*	28 (8.1)	26 (7.6)	<0.001*
Pyrexia	44 (6.8)	24 (3.7)	0.002*	87 (12.3)	38 (5.4)	0.002*	122 (35.5)	101 (29.4)	0.033*
Hyperpyrexia	-	-	-	2 (0.3)	-	<0.001*^	1 (0.3)	1 (0.3)	1^
At least one unsolicited ADR, n (%)	607 (94.0)	553 (85.6)	1	413 (58.4)	254 (35.9)	<0.001*^	304 (88.4)	265 (77.0)	0.839
At least one AESI, n (%)	1 (0.2)	2 (0.3)	<0.001*^	-	1 (0.1)	0.617	-	-	-

At least one serious ADR, n (%)	1 (0.2)	3 (0.5)	<0.001*^	4 (0.6)	1 (0.1)	<0.001*	3 (0.9)	4 (1.2)	0.185

Abbreviations: ADR = adverse drug reaction, MedDRA=Medical Dictionary for Regulatory Activities; PT=preferred term; * = statistically significant; ^= Fisher's exact test

Supplementary Table S4. Frequency of reported local and systemic solicited ADRs following the booster dose, stratified by vaccine brands, in people with SARS-CoV-2 infection vs. matched control

	Booster dose					
	Comirnaty (BioNTech/Pfizer) N= 521 (%)			Spikevax (Moderna) N= 376 (%)		
	Prior SARS-CoV-2 infection	Matched control	p-value	Prior SARS-CoV-2 infection	Matched control	p-value
At least one ADR, n (%)	334 (64.1)	317 (60.8)	0.306	278 (73.9)	249 (66.2)	0.025*
At least one solicited ADR n (%)	317 (60.8)	302 (58)	0.377	270 (71.8)	244 (64.9)	0.05*
**Local solicited ADR (MedDRA PT)**						
Injection site erythema	26 (5)	24 (4.6)	0.885	20 (5.3)	33 (8.8)	0.087
Injection site haematoma	12 (2.3)	9 (1.7)	0.659	13 (3.5)	12 (3.2)	1.000
Injection site induration	3 (0.6)	1 (0.2)	0.616	2 (0.5)	3 (0.8)	1.000
Injection site inflammation	75 (14.4)	69 (13.2)	0.654	61 (16.2)	69 (18.4)	0.500
Injection site pain	210 (40.3)	191 (36.7)	0.252	177 (47.1)	165 (43.9)	0.420
Injection site pruritus	17 (3.3)	11 (2.1)	0.164	11 (2.9)	16 (4.3)	0.433
Injection site reaction	1 (0.2)	-	1.000	-	-	
Injection site swelling	58 (11.1)	73 (14)	0.191	65 (17.3)	68 (18.1)	0.848
Injection site warmth	44 (8.4)	30 (5.8)	0.117	32 (8.5)	37 (9.8)	0.613
**Systemic solicited ADR (MedDRA PT)**						
Arthralgia	78 (15)	53 (10.2)	0.024*	55 (14.6)	56 (14.9)	1.000
Chills	115 (22.1)	59 (11.3)	<0.001*	102 (27.1)	91 (24.2)	0.404
Fatigue	194 (37.2)	163 (31.3)	0.050	158 (42)	137 (36.4)	0.135
Headache	125 (24)	115 (22.1)	0.508	131 (34.8)	114 (30.3)	0.213
Malaise	131 (25.1)	99 (19.0)	0.020*	114 (30.3)	94 (25)	0.121
Myalgia	136 (26.1)	111 (21.3)	0.080	114 (30.3)	106 (28.2)	0.575
Nausea	45 (8.6)	40 (7.7)	0.651	47 (12.5)	26 (6.9)	0.013*

Body_temperature_increased	42 (8.1)	23 (4.4)	0.021*	30 (8)	28 (7.4)	0.891
Pyrexia	62 (11.9)	41 (7.9)	0.037*	69 (18.4)	59 (15.7)	0.383
Hyperpyrexia	1 (0.2)	1 (0.2)	1.000	-	-	
At least one unsolicited ADR, n (%)	87 (16.7)	114 (21.9)	0.041*	72 (19.2)	57 (15.2)	0.176
At least one AESI, n (%)				1 (0.3)		1
At least one serious ADR, n (%)	2 (0.4)	1 (0.2)	1			

Abbreviations: ADR = adverse drug reaction, MedDRA=Medical Dictionary for Regulatory Activities; PT=preferred term; * = statistically significant; ^= Fisher's exact test

2 vaccinees who reported an unknown vaccine brand and 3 Vaxzevria were excluded

Supplementary Table S5. Frequency of reported local and systemic solicited ADRs following the first vaccination cycle of any vaccine, stratified by gender, in people with SARS-CoV-2 infection vs. matched control

	First vaccination cycle											
	First dose						Second dose					
	Males N= 991 (%)			Females N= 2,895 (%)			Males N= 470 (%)			Females N= 1,229 (%)		
	Prior SARS-CoV-2 infection	Matched control	p-value	P Prior SARS-CoV-2 infection	Matched control	p-value	Prior SARS-CoV-2 infection	Matched control	p-value	Prior SARS-CoV-2 infection	Matched control	p-value
At least one ADR, n (%)	778 (78.5)	690 (69.6)	<0.001*	2,702 (93.4)	2,593 (89.6)	<0.001*	373 (79.4)	318 (67.7)	0.407	1165 (94.8)	1,110 (90.3)	0.811
At least one solicited ADR, n (%)	580 (58.5)	566 (57.1)	1	2,202 (76.1)	2,379 (82.2)	0.462	242 (51.5)	187 (46.0)	0.202	791 (64.4)	740 (60.2)	0.645
**Local solicited ADRs (MedDRA PT)**												
Injection site erythema	31 (3.1)	26 (2.6)	0.237	249 (8.6)	180 (6.2)	0.059	16 (3.4)	10 (2.1)	<0.001*	85 (6.9)	98 (8)	0.812
Injection site haematoma	32 (3.2)	26 (2.6)	0.186	132 (4.6)	139 (4.8)	0.239	9 (1.9)	13 (2.8)	<0.001*	46 (3.7)	35 (2.8)	0.239
Injection site induration	4 (0.4)	4 (0.4)	0.860^	23 (0.8)	38 (1.3)	0.012*	1 (0.2)	0 (0.0)	1^	4 (0.3)	2 (0.2)	<0.001*
Injection site inflammation	80 (8.1)	62 (6.3)	0.305	605 (20.9)	490 (16.9)	0.364	35 (7.4)	21 (4.5)	0.019*	184 (15)	156 (12.7)	0.303
Injection site pain	281 (28.4)	291 (29.4)	1	1,315 (45.4)	1,291 (44.6)	0.948	110 (23.4)	76 (16.2)	<0.001*	389 (31.7)	363 (29.5)	0.663
Injection site pruritus	12 (1.2)	11 (1.1)	0.188	115 (4)	99 (3.4)	<0.001*	5 (1.1)	2 (0.4)	<0.001*	38 (3.1)	28 (2.3)	0.092
Injection site reaction	1 (0.1)	2 (0.2)	<0.001*^	2 (0.1)	1 (0)	<0.001*^	-	-	-	-	-	1
Injection site swelling	83 (8.4)	62 (6.3)	0.265	521 (18)	408 (14.1)	0.4	37 (7.9)	16 (3.4)	<0.001*	153 (12.4)	119 (9.7)	0.016
Injection site warmth	36 (3.6)	28 (2.8)	0.168	386 (13.3)	301 (10.4)	0.146	17 (3.6)	12 (2.6)	0.003*	120 (9.8)	126 (10.3)	0.737
**Systemic solicited ADRs (MedDRA PT)**												
Arthralgia	99 (10.0)	70 (7.1)	0.187	641 (22.1)	517 (17.9)	0.361	33 (7)	31 (6.6)	0.857	135 (11.0)	98 (8)	<0.001*
Chills	175 (17.7)	117 (11.8)	<0.001*	1100 (38)	971 (33.6)	<0.001*	73 (15.5)	39 (8.3)	<0.001*	202 (16.4)	184 (15)	0.396
Fatigue	274 (27.6)	255 (25.7)	0.564	1405 (48.5)	1365 (47.2)	1	120 (25.5)	93 (19.8)	<0.001*	406 (33)	377 (30.7)	0.594
Headache	265 (26.7)	186 (18.8)	0.045*	1374 (47.5)	1295 (44.7)	0.731	93 (19.8)	68 (14.5)	0.403	369 (30)	331 (26.9)	0.707
Malaise	257 (25.9)	176 (17.8)	0.024*	1342 (46.4)	1188 (41.1)	0.228	124 (26.4)	84 (17.9)	<0.001*	382 (31.1)	313 (25.5)	0.225

Myalgia	282 (28.5)	259 (26.1)	0.433	1365 (47.2)	1245 (43)	0.410	92 (19.6)	88 (18.7)	<0.001*	320 (26)	286 (23.3)	0.307
Nausea	83 (8.4)	56 (5.7)	0.115	648 (22.4)	582 (20.1)	0.563	29 (6.2)	21 (4.5)	0.142	160 (13)	126 (10.3)	0.030
Body temperature increased	41 (4.1)	24 (2.4)	0.465	118 (4.1)	129 (4.5)	0.376	16 (3.4)	9 (1.9)	<0.001*	47 (3.8)	38 (3.1)	0.362
Pyrexia	144 (14.5)	100 (10.1)	<0.001*	894 (30.9)	702 (24.3)	0.36	56 (11.9)	29 (6.2)	0.012*	197 (16)	134 (10.9)	0.005
Hyperpyrexia	3 (0.3)	0 (0.0)	<0.001*^	19 (0.7)	15 (0.5)	0.122	1 (0.2)	-	1^	2 (0.2)	1 (0.1)	<0.001*

Abbreviations: ADR = adverse drug reaction, MedDRA=Medical Dictionary for Regulatory Activities; PT=preferred term; * = statistically significant; ^= Fisher's exact test

Supplementary Table S6. Frequency of reported local and systemic solicited ADRs following the booster dose of any vaccine, stratified by gender for people with SARS-CoV-2 infection and the matched control

	**Booster dose**					
	**Males N= 312 (%)**			**Females N= 590 (%)**		
	**Prior SARS-CoV-2 infection**	**Matched control**	**p-value**	**Prior SARS-CoV-2 infection**	**Matched control**	**p-value**
At least one ADR, n (%)	195 (62.5)	160 (51.3)	0.364	421 (71.4)	407 (67.0)	1
At least one solicited ADR, n (%)	185 (59.3)	156 (50.0)	<0.001*	406 (68.8)	391 (66.3)	<0.001*
**Local solicited ADRs (MedDRA PT)**						
Injection site erythema	8 (2.6)	6 (1.9)	0.603	38 (6.4)	391 (66.3)	0.014*
Injection site haematoma	3 (1)	2 (0.6)	0.0367*	22 (3.7)	51 (8.6)	<0.001*
Injection site induration	1 (0.3)	1 (0.3)	1^	4 (0.7)	19 (3.2)	<0.001*^
Injection site inflammation	29 (9.3)	22 (7.1)	0.207	107 (18.1)	3 (0.5)	<0.001*^
Injection site pain	112 (35.9)	87 (27.9)	<0.001*	276 (46.8)	116 (19.7)	<0.001*
Injection site pruritus	2 (0.6)	-	0.479	26 (4.4)	269 (45.6)	0.162
Injection site reaction	-	-	-	1 (0.2)	27 (4.6)	<0.001*^
Injection site swelling	29 (9.3)	23 (7.4)	0.278	94 (15.9)	-	<0.001*^
Injection site warmth	12 (3.8)	7 (2.2)	<0.001*	65 (11)	119 (20.2)	<0.001*
**Systemic solicited ADRs (MedDRA PT)**						
Arthralgia	36 (11.5)	26 (8.3)	0.226	97 (16.4)	83 (14.1)	0.686
Chills	64 (20.5)	35 (11.2)	<0.001*	155 (26.3)	115 (19.5)	<0.001*
Fatigue	102 (32.7)	73 (23.4)	<0.001*	253 (42.9)	227 (38.5)	0.002*
Headache	63 (20.2)	54 (17.3)	<0.001*	194 (32.9)	175 (29.7)	0.651
Malaise	66 (21.2)	55 (17.6)	0.648	181 (30.7)	138 (23.4)	0.116
Myalgia	74 (23.7)	59 (18.9)	0.624	179 (30.3)	159 (26.9)	0.749
Nausea	14 (4.5)	6 (1.9)	<0.001*	78 (13.2)	60 (10.2)	0.423
Body temperature increased	26 (8.3)	12 (3.8)	<0.001*	47 (8.0)	39 (6.6)	0.578

Pyrexia	52 (16.7)	33 (10.6)	0.147	80 (13.6)	67 (11.4)	0.159
Hyperpyrexia	-	-	-	1 (0.2)	1 (0.2)	1

Abbreviations: ADR = adverse drug reaction, MedDRA=Medical Dictionary for Regulatory Activities; PT=preferred term; * = statistically significant, ^= Fisher's exact test

Supplementary Table S7. Frequency of people with prior SARS-CoV-2 infection reporting at least one ADR, stratified by different timeframes between SARS-CoV-2 infection and vaccine administration, and vaccine dose

		0-90 days	91-180 days	181-360 days	>360 days	*p-value*
First dose	Total#	933	1,005	860	866	0.076
	At least one ADR, n (%)	864 (92.6)	899 (89.5)	784 (91.2)	778 (89.8)	
Second dose	Total#	336	343	387	523	0.128
	At least one ADR, n (%)	308 (91.7)	309 (90.1)	363 (93.8)	475 (90.8)	
Booster dose	Total#	1	-	-	45	1
	At least one ADR, n (%)	1 (100)	-	-	31 (68.9)	

#Number of patients who answered the specific questions in the baseline

Supplementary Table S8. Frequency of people with prior SARS-CoV-2 infection reporting at least one ADR, stratified by different variants of concern

		Alpha variant	Delta variant	*p-value*
First dose	Total#	3653	39	0.006*
	At least one ADR	3321 (90.9)	30 (76.9)	
Booster dose	Total#	50	36	0.947
	At least one ADR	11 (22.0)	9 (25.0)	

#Number of patients who reported the date of onset of symptoms, * = statistically significant

Supplementary Table S9. Frequency of people with prior SARS-CoV-2 infection reporting at least one ADR, stratified by different severity of symptoms

		No symptoms	Symptoms similar to cold	Many symptoms	Admitted to hospital	*p-value*
First dose	Total#	758	1167	1836	78	<0.001*
	At least one ADR	602 (79.4)	1035 (88.7)	1733 (93.4)	67 (85.9)	
Booster dose	Total#	221	377	281	19	<0.001*
	At least one ADR	123 (55.6)	269 (71.4)	207 (73.6)	16 (84.2)	

#Number of patients who answered the specific questions in the baseline , * = statistically significant

Supplementary Table S10. People with prior SARS-CoV-2 infection stratified by country

	**First vaccination cycle**	**Booster dose**
**Total**	**3,886 (100)**	**902 (100)**
Belgium	10 (0.2)	-
France	216 (5.5)	351 (38.9)
Italy	139 (3.6)	226 (25.1)
Ireland	-	14 (1.6)
The UK	51 (1.3)	147 (16.3)
The Netherlands	3,434 (88.4)	-
Portugal	4 (0.1)	13 (1.4)
Romania	13 (0.3)	67 (7.4)
Slovakia	13 (0.3)	-
Spain	4 (0.1)	54 (6.0)
Switzerland	2 (0.1)	30 (3.3)
